# A Feasible Web-Conference-Style Remote Simulation using Demonstration Video Clips in Anaesthesia under the COVID-19 Outbreaks: A Preliminary Survey Study

**DOI:** 10.4274/TJAR.2023.221166

**Published:** 2023-08-18

**Authors:** Taiki Kojima, Yuta Kawatsu

**Affiliations:** 1 Department of Anaesthesiology, Aichi Children’s Health and Medical Center, Aichi, Japan; 2 Division of Comprehensive Pediatric Medicine, Nagoya University Graduate School of Medicine, Aichi, Japan

**Keywords:** Anaesthesia, COVID-19, remote, simulation, web-conference-style

## Abstract

**Objective:**

The Coronavirus disease-2019 (COVID-19) outbreak has deprived simulation-based medical education for health care workers. Attendees are strictly prohibited to converge at a simulation training venue because of the COVID-19 outbreaks. To address this issue, we developed a web-conference-style remote simulation program using demonstration video clips. This report introduced the method and described participants’ satisfaction.

**Methods:**

This preliminary survey study evaluated learners’ satisfaction in multiple institutions. The Satisfaction Scale Questionnaire with High-Fidelity Clinical Simulation (SSHF) by a 5-degree scale was used to evaluate participants’ satisfaction. The survey was conducted immediately after completing the simulation sessions.

**Results:**

Ten (100%) participants (7 anaesthesia individuals, 2 anaesthesia residents and 1 nurse anaesthetist) from nine institutions responded to the survey. All median values of the satisfaction scores were ≥4.0, whereas the median values of scores for environmental fidelity and psychological insecurity were 3.5 and 3.0, respectively (*P*=0.005).

**Conclusion:**

A web-conference-style remote simulation using demonstration video clips is a feasible method for conducting simulation-based medical education under COVID-19 that showed high satisfaction scores. Further, additional studies are required to explore the internal and external validity and the effectiveness of mastery learning.

**Main Points**

• A web-conference-style complete remote simulation using demonstration video clips can be a feasible approach under the coronavirus disease-2019 outbreaks.

• A web-conference-style complete remote simulation using video clips focused on teaching the two aspects of team dynamics and medical management.

• The overall satisfaction scores of the participants for a complete remote simulation using video clips were generally high.

## Introduction

Simulation-based medical education (SBME) is commonly performed as an effective training method to enhance individual clinical skills, judgment, team dynamics and resource management.^1^ However, the Coronavirus disease-2019 (COVID-19) outbreaks have become a major obstacle in providing manikin-based SBME practices because of social distancing as participants must travel from different places and gather at a training venue (e.g. simulation centre or hospital). Thus, a new SBME technique that does not require learners to congregate in one place is desired.

Even under the COVID-19 outbreaks, remote simulation using telecommunication technologies can be a viable method in providing SBME since the learners can remotely participate in the simulation sessions via the internet.^2^ However, a remote simulation was originally designed to remotely provide facilitation from the outside of the simulation site for the learners gathered at the simulation venue to perform a hands-on training. Therefore, the proposed method for remote simulation is not ideal to conduct SBME owing to the potential risks of COVID-19 outbreaks.

To address this issue, we modified the structure to prevent learners from gathering at the simulation venue, and they, along with the facilitators, participated in the SBME sessions remotely via the internet. The learners were unable to practice on-site simulation scenarios. Thus, we created demonstration video clips that were used as materials for discussion-based learning.

This report presents the web-conference-style remote simulation using demonstration video clips and the survey results of participants’ perceptions.

## Methods

### Study Design, Participants

This was a preliminary survey study on anaesthesia providers, who participated in a web-conference-style remote simulation programme in October, 2021. This study obtained the approval of the Local Institutional Ethics Committee of Aichi Children’s Health and Medical Center (approval number: 2021053, September 11, 2021).

### Course of Web-Conference-Style Remote Simulation

**Figure 1:**
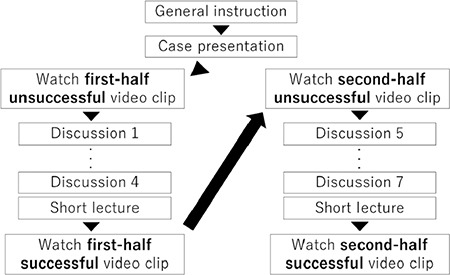
Flowchart of the web-conference-style simulation session of the difficult airway scenario.

Learners and facilitators remotely participated in the online conference sessions. The remote simulation was structured into a 50 min simulation session each for two scenarios (i.e. cardiac arrest and difficult airway). Two facilitators experienced in *in situ* anaesthesia simulation separately conducted each simulation session. After a brief explanation of the cases in each scenario, the learners watched a video clip of an unsuccessful demonstration. Further, the participants analysed the actions of the actors in the video clip in terms of teamwork/communication and medical expertise along with the prepared questions that were created based on the pre-determined checklists (Table 1). Between each discussion, the essential knowledge of teamwork/communication and medical knowledge was addressed by a brief didactic lecture series to enhance learners’ understanding. After the discussion and a brief lecture, learners watched a successful demonstration video clip and discussed improvements in the unsuccessful demonstration video clip (Figure 1). Regarding medical knowledge, participants analysed the Pediatric Advanced Life Support (PALS) algorithm for cardiac arrest and American Society for Pediatric Anesthesia (SPA) critical events checklists for difficult airways in real-life clinical scenarios via demonstration video clips that re-enacted clinical scenes.

### Simulation Scenarios

Two distinct paediatric critical scenarios during general anaesthesia (i.e. cardiac arrest and difficult airway) were created. These simulation scenarios were designed to cover the pre-determined checklists of the learners’ expected actions for effective management in each critical event, and their expected actions were classified into two major categories: teamwork/communication and medical knowledge. The teamwork/communication checklists included team dynamics and interpersonal communication skills (i.e. role assignment, sharing mental models/critical information, closed-loop communication), whereas, medical knowledge checklists were developed based on the critical event checklists of SPA and PALS.^3,4^ The learners were expected to perform all of the pre-determined tasks in the checklists.

### Demonstration Video Clips for Successful and Unsuccessful Instances

**Figure 2:**
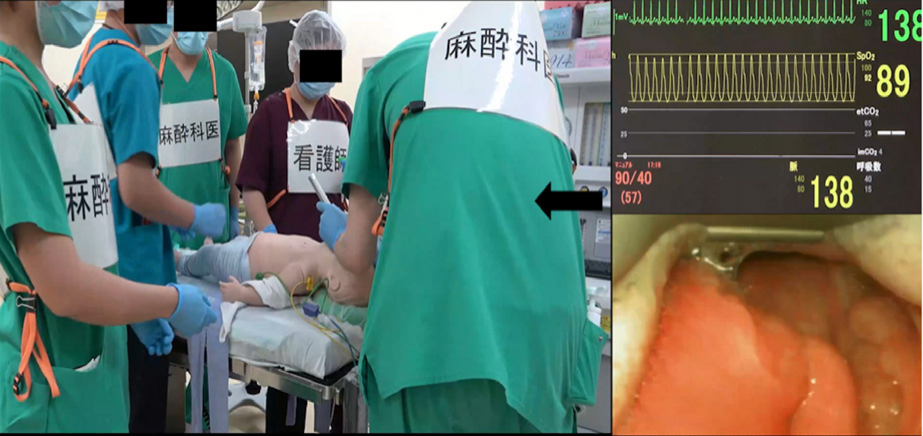
Screenshot of the demonstration video clip in the difficult airway scenario. An actor anaesthesiologist (black arrow) was attempting endotracheal intubation. Left, the scene in the scenario; right upper, vital signs; right lower, a picture of the laryngeal exposure.

Two types of demonstration video clips were developed for successful and unsuccessful instances for each scenario (i.e., cardiac arrest and difficult airway) (Figure 2). For successful instances, actors playing the roles of anaesthesiologists and operating room nurses implemented the pre-determined tasks in the checklists regarding teamwork/communication and medical knowledge. In contrast, these were not sufficiently implemented in the unsuccessful instances. The following three major components were incorporated into the successful instances, while not into the unsuccessful instances: 1) crisis management (i.e., situation awareness, resource management), 2) team dynamics (i.e., statement of emergency, sharing mental models, role assignment, frequent mutual verbal communication, closed-loop communication), 3) medical management (i.e., background knowledge, algorithm implementation). These video clips mainly focus on learning how to prevent human errors in a resuscitation scene. We applied the anaesthetists’ non-technical skills scoring tool.^5^ This tool has four major categories (i.e., task management, team working, situation awareness, and decision making) with three to four subcategories rated from 1 to 4. We selected most components from the tool based on the capability to learn them in discussion-style, knowledge-based learning.

The demonstration video clips show: 1) the scene of implementing the pre-determined tasks by anaesthesiologists and operating room nurses, 2) the monitor displaying vital signs of the manikin and 3) subtitles describing the conversation among actors in the demonstration scenes. In advance, to create demonstration video clips for successful and unsuccessful instances, the demonstration scenes by actors in the operating rooms and the monitor of manikin’s vital signs were recorded simultaneously using video cameras. The recorded video files (i.e. MP4 format) of demonstration scenes were edited into the final demonstration video clips with subtitles of actors’ conversation using a video editing software (Filmora X^®^, Wondershare, Shenzhen, China) (Figure 2).

### Two Parts of Demonstration Video Clips

The duration of both final video clips was ~7 min, which is divided into two parts because of the progression of each scenario. The first half focused on the situation awareness regarding the patient’s sudden deterioration and the team dynamics for early transition to initial treatments (e.g. declaration of the patient’s deterioration, call for help, role assignment and initiation of cardiopulmonary resuscitation). In the second half, the patient’s condition deteriorated more, such that individual clinical skills, judgement, team dynamics and resource management were focused on situations requiring advanced medical management (e.g. implementation of PALS and difficult airway management algorithms, closed-loop communication, recap the situation, request necessary resources).

### Data Collection

Anonymous survey forms were distributed to learners after completing the simulation sessions. The data regarding the characteristics of learners, including age, sex, post-graduate year and simulation experience (i.e. none, limited, moderate or extensive) were collected. The Satisfaction Scale Questionnaire with High-Fidelity Clinical Simulation (SSHF) was used to determine the participants’ satisfaction with the remote simulation sessions.^4,6^ SSHF is a validated evaluation tool of simulation sessions that originally comprised 38 closed questions evaluated by a 5-degree Likert scale, integrating into eight domains: 1) simulation utility, 2) scenario cases and applications, 3) team communication, 4) self-reflection on performance, 5) increase in self-confidence, 6) connection between theory and practice, 7) facilities and equipment and 8) negative aspects of the simulation session.^4,6^ The two questions in the original SSHF: 1) were removed because of their irrelevance to the simulation scenarios in the current study since learners did not have the opportunities to improve their communication skills with the patient and family members during general anaesthesia in the operating room.

### Statistical Analysis

The primary investigator collected the survey forms and summarised the descriptive statistics of the results. The summary statistics were described in median [interquartile range (IQR)] or number (%). The median values of the score in each questionnaire item were compared using the Kruskal-Wallis test. All summary statistics were calculated using STATA 17.0 (StataCorp, College Station, TX, USA).

## Results

The simulation sessions involved 10 learners (7 anaesthesia individuals, 2 anaesthesia residents and 1 nurse anaesthetist) from nine institutions. The response rate was 100%.

### Learners’ Characteristics

The following are the learner’s characteristics: age, median (IQR), 30 (27-33); female, 5/10 (50%); previous experiences of manikin-based simulation, none 4/10 (40%); limited 4/10 (40%); moderate 2/10 (20%).

### Learners’ Satisfaction

The median values of the scores of learners’ satisfaction for the remote simulation were ≥4.0, except for the median values for the two items “the reality of facilities and equipment” (3.5) and “I was thrown off balance during some of the cases” (3.0) (Table 2). There was a significant difference among the scores (*P*=0.005).

## Discussion

This was the preliminary survey study to conduct a web-conference-style remote simulation using demonstration video clips and evaluate the perceptions of the learners. The overall satisfaction scores of the learners were generally high, whereas the scores for environmental fidelity and psychological safety were relatively lower compared with the other items.

The COVID-19 outbreaks have enforced medical educators to conduct SBME using virtual platforms. A remote simulation in operating rooms can now be a solution to provide *in situ* simulation training for anaesthesia providers to avoid participants from different sites assembling in a single venue. In the remote simulation, two potential methods exist: 1) trainees assemble in the operating room to begin a simulation scenario as a resuscitation team and the facilitator remotely teaches learners via the internet and 2) faculty members assemble in the operating room to proceed with a simulation scenario as a resuscitation team and learners remotely lead the team via the internet, However, proceeding a simulation scenario requires several participants (learners or faculty members), in both methods, to conduct the simulation scenario in each simulation training session. Therefore, we developed a web-conference-style remote simulation technique using video clip materials that allow all participants (i.e., faculty members and learners) to independently participate in the simulation sessions.

The present study showed high learners’ satisfaction despite a lack of opportunities for learners to physically conduct the simulation scenario in the operating room. However, scores for environmental fidelity and psychological safety were relatively lower than that for other domains. These results are attributed to several explanations. First, learners participated in the simulation session to engage in the discussion for improving situation awareness, team dynamics, medical knowledge and clinical judgement from clinical scenes in the video clip materials that they watched on their computer screens. The absence of in-situ training in the operating room might have unsatisfied learners regarding environmental fidelity. Advanced virtual technologies (e.g. virtual reality) providing realistic remote simulation are considered a solution for providing sufficient environmental fidelity to learners participating in the remote simulation via the internet. Second, our simulation programme was developed as a practical session at an academic conference. Therefore, the simulation sessions that can endanger the learners’ psychological safety because these simulation sessions were watched by other audiences via the online video-sharing platform. Our web-conference-style remote simulation can be performed within a closed community enabling facilitators to provide a psychologically safe environment for learners.

There are several strengths in our web-conference-style remote simulation technique, except for minimising the risk of COVID-19 outbreaks. First, compared with the conventional manikin-based simulation, this web-conference style simulation can accommodate more participants. Despite the number of roles in a simulation scenario being limited (e.g. leader, chest compressor and recorder), the current new remote simulation is performed in a discussion-based style, which allows numerous individuals to participate simultaneously via the internet. Second, the participants can repeatedly review the performance of the actors by comparing successful and unsuccessful instances. Comparisons of failed and ideal instances can clarify issues happening in real-life critical situations and provide solutions in that clinical context.

### Study Limitations

There are several significant limitations in this study. First, a small sample size was used in this preliminary survey study. However, this new method can accommodate more people compared with conventional in-situ manikin-based simulation. Thus, further prospective studies with larger sample sizes are considered feasible. Second, most participants were unfamiliar with all components (crisis management, team dynamics, and medical management) due to a lack of knowledge and skills. The unfamiliarity with the simulation training could result in high satisfaction scores for the current simulation program. However, our simulation program did not provide opportunities for skill training. This simulation program mainly focused on learning the knowledge aspects of the major components (i.e., crisis management, team dynamics, medical management) for successful resuscitation because a COVID-19 surge eliminated learning opportunities for novice trainees regarding team resuscitation. Third, the mastery of learning teamwork/communication and medical knowledge was not evaluated. Fourth, this remote simulation was performed in paediatric anaesthesia scenarios, which require further validation assessment in other clinical scenarios (e.g., emergency department, general paediatrics). Finally, this study does not dismiss the significance of manikin-based simulation procedures. However, in critical situations, the currently modified remote simulation cannot substitute manikin-based simulation practices to improve situation awareness and review the local response systems. Further, this new method is situated between the knowledge and practice levels in Miller’s pyramid, which facilitates learners to develop a realistic image of appropriate actions to maximise the team dynamics during critical events.^7^

## Conclusion

Under the COVID-19 outbreaks, a web-conference-styled remote simulation, which uses demonstration video clips can be a feasible approach for simulation-based education. Further additional prospective investigations that evaluate the effectiveness of mastery learning and validation study are required.

## Figures and Tables

**Table 1:**
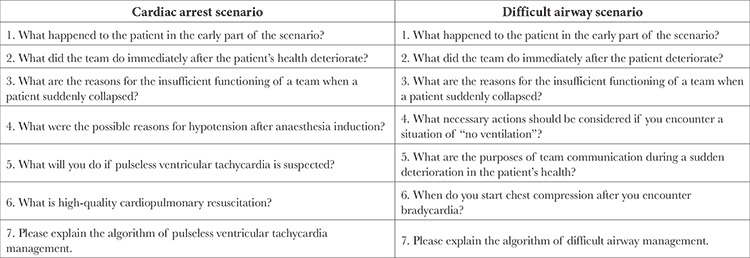
Questions that were Discussed by Learners During the Simulation Sessions of Cardiac Arrest and Difficult Airway Scenarios

**Table 2:**
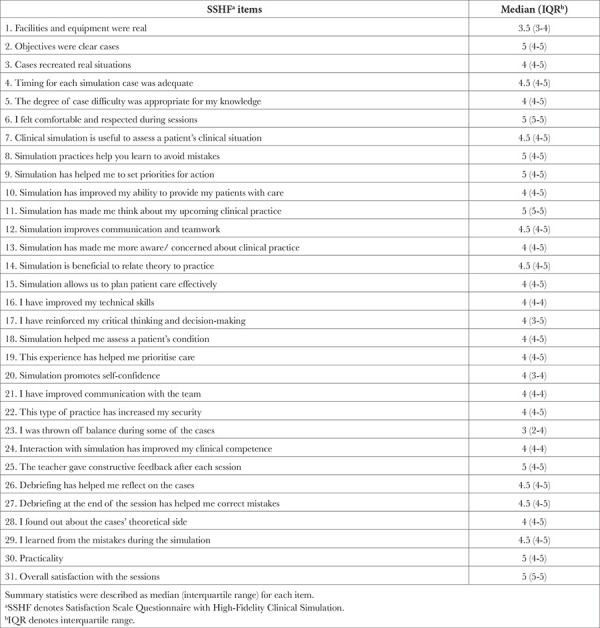
Median and Interquartile Values of SSHFa Items (1-19) in Participants (n = 10)
